# Assessing the impact of open-circuit voltage estimation methods on UKF performance for lithium-ion battery SOC and SOH estimation

**DOI:** 10.1038/s41598-026-38846-4

**Published:** 2026-02-06

**Authors:** M. Mikhak-Beyranvand, M. Salehi, M. A. Mohammadkhani

**Affiliations:** Department of Electrical Engineering, Technical and Vocational University (TVU), Tehran, Iran

**Keywords:** State of charge (SOC), Unscented kalman filter (UKF), Open-Circuit voltage (OCV), Low-Current method (LC), Incremental current method (IC), Internal resistance (R_0_), Energy science and technology, Engineering

## Abstract

Accurate estimation of State of Charge (SOC) and State of Health (SOH) in lithium-ion batteries is critical for effective Battery Management Systems (BMS). A key factor in enhancing estimation accuracy is the appropriate selection of the Open-Circuit Voltage (OCV-SOC) model. This study investigates two prevalent methods for OCV modeling: the Low-Current (LC) method and the Incremental Current (IC) method, and evaluates their impact on the performance of the Unscented Kalman Filter (UKF) for SOC estimation and instantaneous internal resistance (R_0_) tracking. In this work, SOH is primarily assessed through R_0_​, which is adopted as the main degradation indicator under dynamic operating conditions. The dynamic model employed is an Equivalent Circuit Model (ECM) of a lithium-ion battery, with estimations conducted using real-world driving data (Federal Urban Driving Schedule, FUDS). The results demonstrate that the IC method, due to its higher local resolution in the OCV modeling, achieves faster convergence and higher estimation accuracy for SOC, particularly when the initial SOC is subject to error. In contrast, the LC method, despite its experimental simplicity, exhibits significant errors in the early stages of simulation due to excessive smoothing of the OCV curve. Furthermore, a comparison of R_0_ trends reveals that the IC method provides a more consistent and stable estimation, whereas the LC method shows greater short-term fluctuations and reduced stability. This difference is attributed to the higher accuracy of the OCV curve in the IC method. Ultimately, the choice of the OCV extraction method directly influences the accuracy and stability of SOC estimation and SOH analysis based on R_0_ tracking.

## Introduction

Batteries are critical components of energy storage systems in applications such as electric vehicles, playing a pivotal role in ensuring the reliable performance of these systems. Accurate estimation of SOC and SOH is essential for optimizing performance and enhancing system reliability^1,2^. Recent studies have also highlighted the critical role of accurate SOC estimation in protecting battery systems against abuse factors such as overcharge, over-discharge, thermal stress, and accelerated degradation. In this context, SOC-aware battery management strategies have been shown to significantly enhance operational safety, extend battery lifespan, and improve overall system efficiency^3–5^. SOC represents the available electrical energy in the battery, while SOH indicates the remaining performance capability relative to its initial condition. Over multiple charge-discharge cycles, batteries undergo changes such as capacity degradation, increased R_0_, and cell imbalances, which can impair system performance^6,7^. Since key parameters determining SOC and SOH, such as remaining capacity or R_0_, cannot be directly measured from battery terminals, the development of indirect model-based or data-driven estimation methods is crucial^8^. In this regard, Equivalent Circuit Models (ECMs), Kalman filter-based methods, and machine learning techniques are among the widely used tools in BMS^9,10^.

One of the primary indicators for assessing SOH is the remaining capacity relative to the nominal capacity. Manufacturers and users often define battery lifespan based on the number of charge-discharge cycles. A standard definition specifies that a battery should be replaced when its capacity falls below 80% of its nominal value^11^. However, determining battery lifespan solely based on cycle count is imprecise, as discharge can occur at varying depths, and no standard definition exists for what constitutes a complete cycle^12,13^. SOH can be estimated through the analysis of OCV and R_0_, particularly the series ohmic resistance (R_0_), which plays a critical role in ECMs. R_0_ increases over time with repeated charge-discharge cycles and is directly correlated with capacity fade^14,15^. Studies have shown that R_0_, as a dynamic parameter, can be identified within the framework of Kalman filters, particularly UKF, enabling real-time monitoring of battery aging^16–18^.

A dataset comprising 50 lithium-ion battery cells was evaluated under charge, discharge, and impedance tests at an ambient temperature of 4 °C, sourced from the NASA Ames Prognostics Data Repository^19^. Each cell was charged at a constant current of 1.5 A to 4.2 V and then discharged at a constant current of 1 A until reaching 2 V. The capacity degradation trend was periodically monitored, and capacity was calculated using the following equation:

1 $$\:{C}_{\mathrm{cap}}=\frac{{Q}_{\left[{t}_{1}\mathrm{,\:}{\mathrm{t}}_{2}\right]}}{{{\Delta\:}\mathrm{E}}_{o}}=\frac{{Q}_{\left[{t}_{1}\mathrm{,\:}{\mathrm{t}}_{2}\right]}}{{E}_{0,{t}_{1}}-{E}_{0,{t}_{2}}}$$

where $\:{{\Delta\:}\mathrm{E}}_{o}$ is the open-circuit voltage difference between two charge states, and $\:{Q}_{\left[{t}_{1}\mathrm{,\:}{\mathrm{t}}_{2}\right]}$ is the charge transferred during the time interval [t₁, t₂], obtained by integrating the discharge current. Subsequently, SOH is calculated as the ratio of the current capacity C_cap_ to the nominal capacity C_n_:

2 $$\:\mathrm{SOH=}\frac{{C}_{cap}}{{C}_{n}}$$

**Fig. 1:**
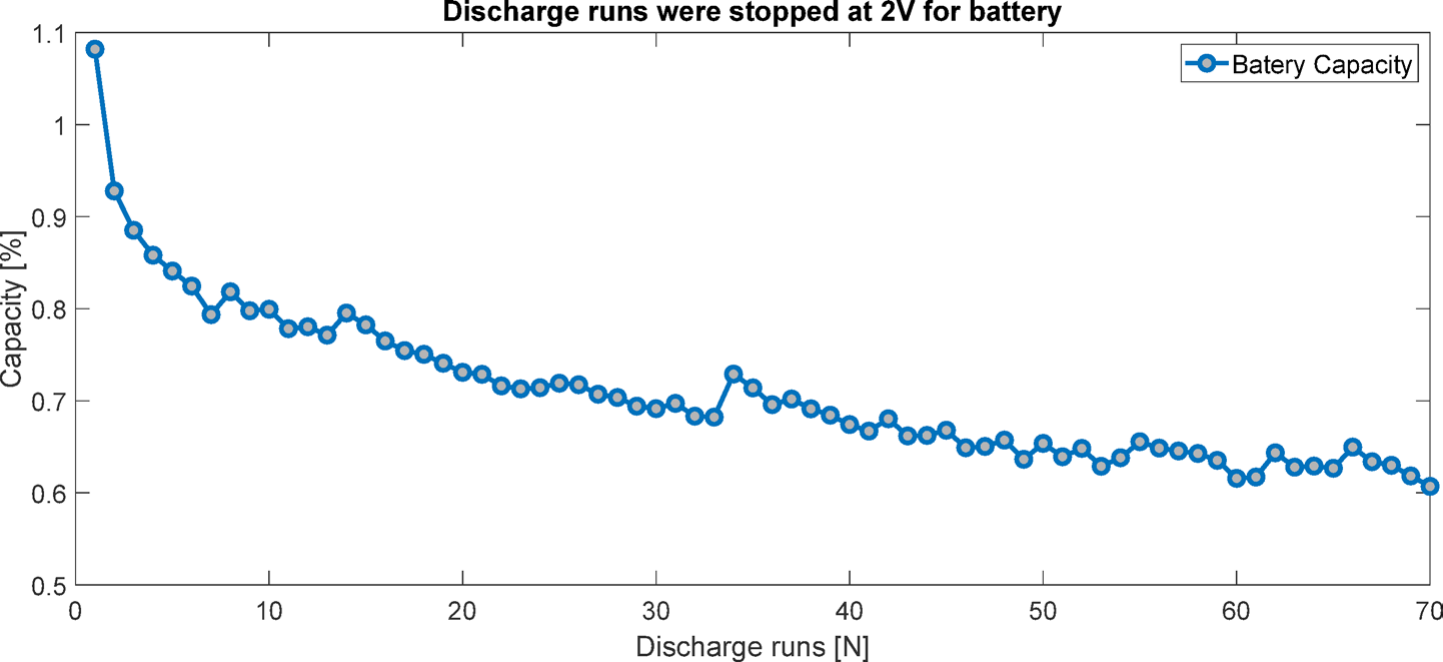
Battery lifespan test for cell 45 after 70 charge-discharge cycles, showing a 30% capacity loss.

Results for cell number 45 indicated a capacity reduction of approximately 30% after about 70 discharge cycles (Fig. 1). These results underscore the suitability of R_0_ as a reliable indicator for SOH estimation in lithium-ion batteries. The increase in R_0_ over charge-discharge cycles is strongly correlated with capacity fade, making it an effective parameter for tracking battery aging trends. By integrating R_0_ estimation within the framework of UKF alongside capacity monitoring, accurate and real-time assessment of SOH can be achieved, as demonstrated in dynamic ECM simulations. This approach, leveraging the sensitivity of R_0_ to battery degradation, provides a robust method for monitoring long-term health trends in BMS^16,17,20–22^.

Accurate modeling of OCV-SOC curve is a critical factor in achieving precise SOC estimation and reliable SOH assessment, serving as the foundation for many model-based estimation methods^17,23,24^. Two common methods for extracting this curve are the LC method and the IC method. The LC method applies low currents (e.g., C/20) and measures voltage under quasi-steady-state conditions, minimizing polarization and resistive voltage drop effects. However, due to excessive smoothing of the OCV curve in regions with steep SOC changes, it may lack sufficient accuracy for dynamic profiles^24,25^. In contrast, the IC method employs current pulses at various SOC levels with extended rest periods, providing more precise data on the battery’s dynamic behavior. Its higher local resolution, particularly in regions with steep OCV slopes, significantly improves the accuracy of SOC and SOH estimation^23,26^. Studies have shown that the IC method, when combined with UKF, offers faster convergence (within fewer than 10 time steps) and lower errors in SOC and R_0_ estimation due to its greater sensitivity to SOC variations^16,18,27^.

This study investigates the impact of the LC and IC methods for OCV-SOC modeling on the performance of the UKF in simultaneously estimating SOC and R_0_ as an indicator of SOH in lithium-ion batteries. Simulations were conducted using FUDS data and OCV tests^28^. Unlike conventional benchmarking studies that primarily rely on steady-state tests or simplified load profiles, the use of the highly dynamic FUDS in this work enables a realistic assessment of UKF convergence behavior, numerical stability, and robustness under frequent current transients representative of real-world BMS operation. This allows the influence of local OCV–SOC resolution on estimation performance to be analyzed beyond static accuracy metrics. The results indicate that the IC method, due to its higher accuracy in representing the OCV curve, achieves SOC estimation with lower RMSE (less than 0.0015) and faster convergence compared to the LC method. The estimation of R_0_ using the IC method also exhibits greater stability, with numerical fluctuations caused by dynamic current variations effectively reduced through a moving average filter. This stability persists even in the presence of initial SOC errors, demonstrating the robustness of the UKF in practical applications. The key contribution of this study is the provision of an integrated framework for evaluating the LC and IC methods in OCV modeling and optimizing SOC and SOH estimation, which can enhance BMS performance in electric vehicles^23,25^. This study offers a practical approach for real-time battery health monitoring, contributing to improved battery lifespan and reliability.

It should be noted that this study focuses on a comparative evaluation of OCV modeling methods within a controlled simulation environment driven by experimental datasets. Direct experimental validation using physically measured SOC and R_0_ is considered an important direction for future research.

## Online SOC and SOH estimation

Real-time estimation of SOC and SOH is critical for the optimal performance of BMS. Unlike offline methods that rely on discharge pulses and extended rest periods, online estimation requires analyzing data under real-world loading conditions. In this study, UKF is employed for simultaneous SOC and SOH estimation under dynamic loading. This approach leverages an ECM with a single RC branch (consisting of series resistance R_0_, polarization resistance Rₚ, and polarization capacitance Cₚ) and precise measurement of OCV to achieve high accuracy in operational conditions^16,18^. The following sections detail the steps for OCV measurement, ECM modeling, and parameter estimation.

### OCV measurement and calculation

OCV is a key parameter in SOC and SOH estimation due to its direct relationship with the battery’s SOC^17,23^. In this study, the LC and IC methods for extracting the OCV-SOC curve are evaluated to assess their impact on SOC and SOH estimation. The data used were sourced from the publicly available CALCE dataset^28^.

#### LC method

The LC method applies low currents (e.g., C/20) to minimize polarization effects and resistive voltage drops, measuring OCV under quasi-steady-state conditions. The implementation steps for this method are as follows^24^:

- *Initial Charge*: The battery is charged at a constant voltage (4.2 V) until the current drops to 0.1 A. This step is brief due to prior charging.
- *Rest Period*: The battery is left without current for approximately 2 h to allow the voltage to stabilize.
- *Constant-Current Discharge*: A current of 0.1 A is applied until the voltage reaches 2.5 V.
- *Second Rest Period*: After stopping the discharge, the resistive voltage drop diminishes, and the voltage stabilizes at 3.16 V.
- *Recharge at C/20 Rate*: The battery is recharged with a current of 0.1 A until reaching the maximum charge voltage. Note that at the start of this step, the voltage exhibits significant dynamic fluctuations, making OCV estimation at low voltages infeasible. Consequently, OCV estimation is performed only for voltages above 3.48 V.

**Fig. 2:**
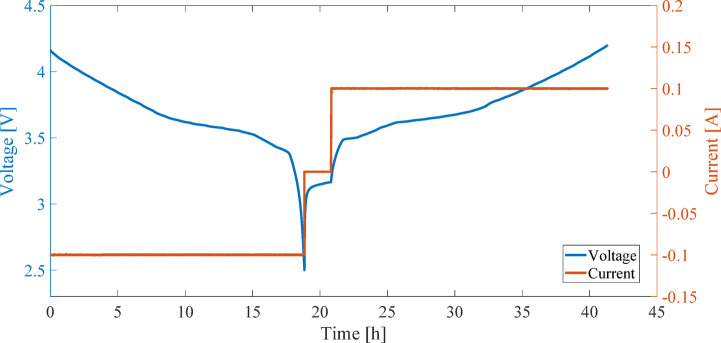
Current and voltage profiles measured during charge and discharge for OCV calculation using the LC method.

**Fig. 3:**
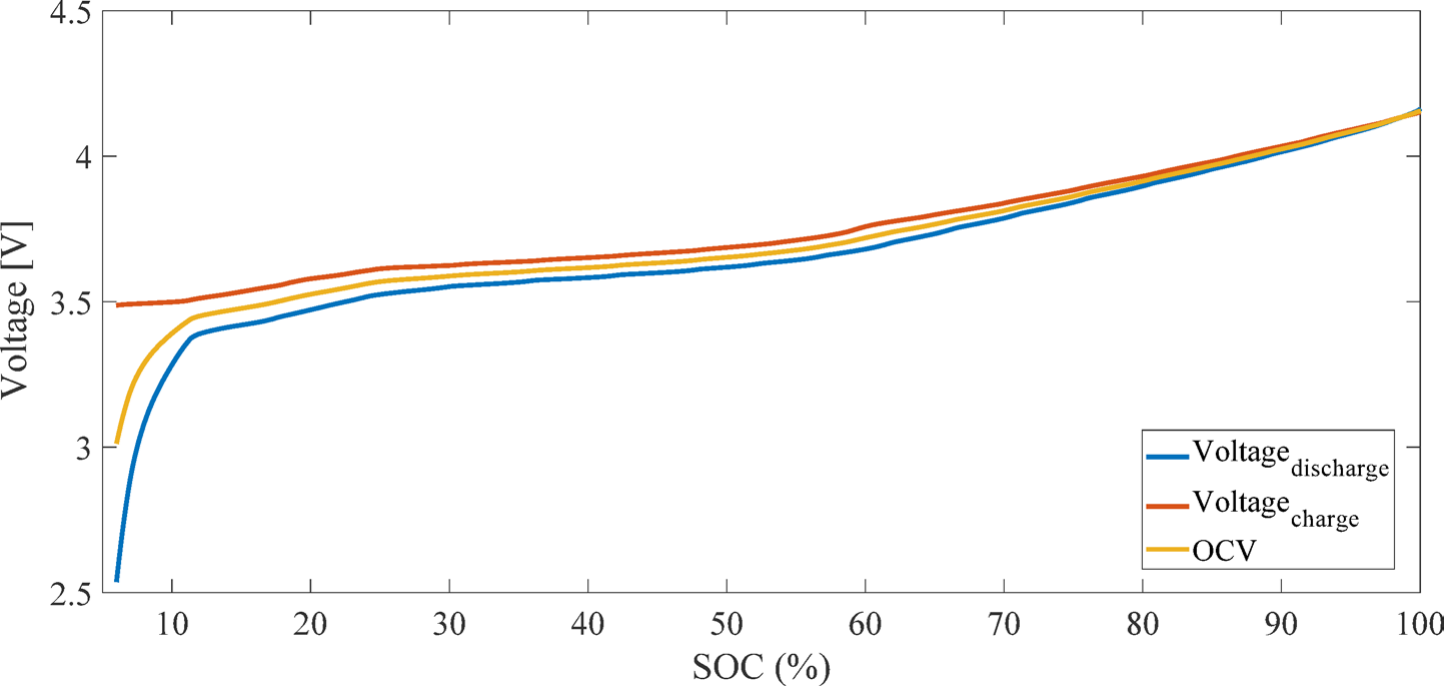
OCV-SOC curve derived from the average of charge and discharge voltages using the LC method.

Figure 2 illustrates the current and voltage profiles measured during the charge and discharge process for calculating OCV using the LC method. Figure 3 presents the OCV-SOC curve derived from the average of charge and discharge voltages using the LC method. Due to the discharge cut-off voltage constraint (2.5 V), the depth of discharge did not exceed 94%, and SOC values below 6% are not represented in the OCV curve^29^.

#### IC method

The IC method applies current pulses at various SOC levels with extended rest periods, providing higher accuracy in regions with steep OCV changes^26^. The implementation steps for this method are as follows:

- *Full Charge*: The battery is charged to 100% SOC.
- *Pulse Discharge*: Negative current pulses (C/2) are applied in 10% SOC increments, with a rest period after each pulse to allow voltage stabilization.
- *Recharge*: After reaching the lower SOC limit, a similar process with positive current pulses is performed to charge the battery back to full SOC.
- *OCV Calculation*: The OCV-SOC curve is obtained by averaging and linearly interpolating the pulse data points.

**Fig. 4:**
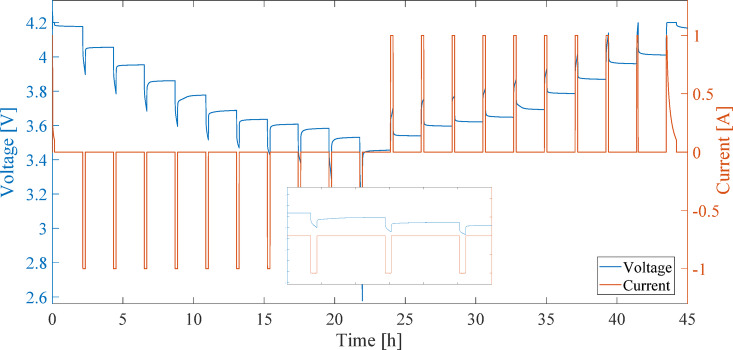
Current and voltage profiles measured during the charge and discharge process for calculating OCV using the IC method.

**Fig. 5:**
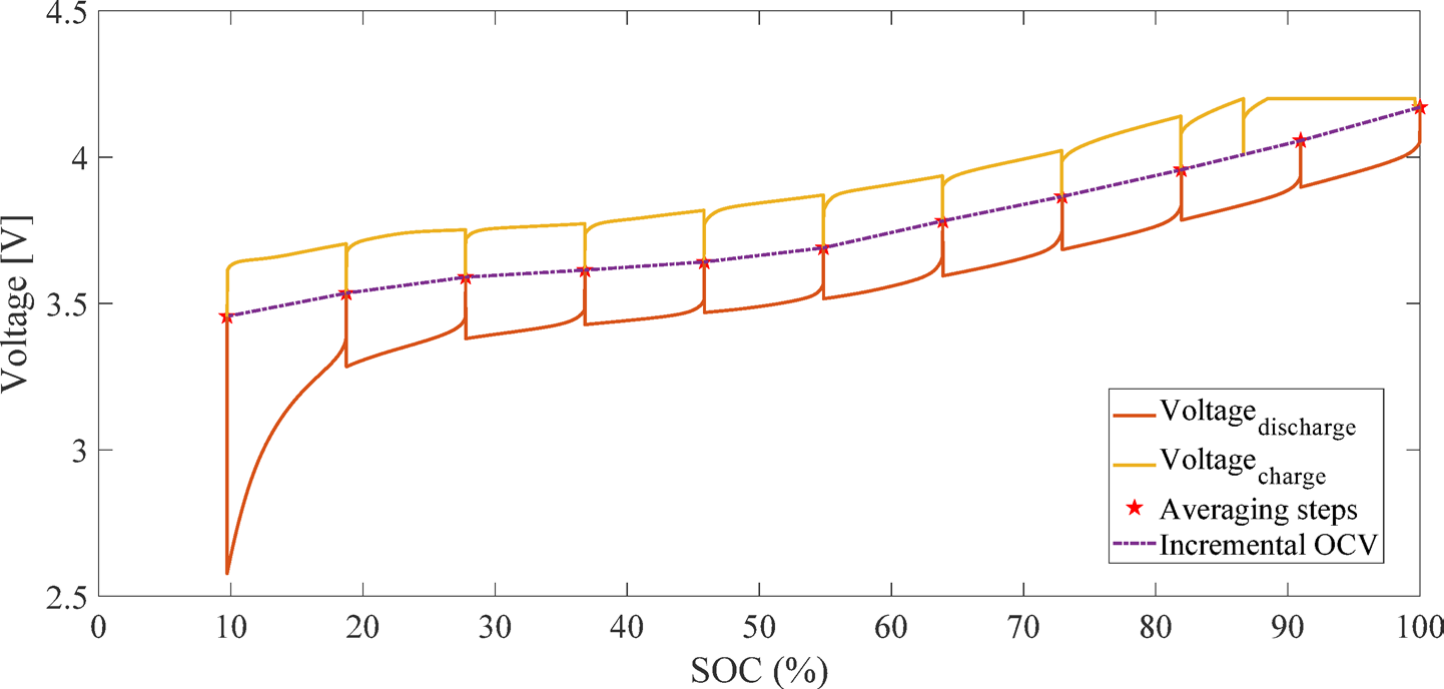
OCV-SOC curve derived from the IC method.

Figure 4 illustrates the current and voltage profiles measured during the charge and discharge process for calculating OCV using the IC method. Figure 5 presents the OCV-SOC curve derived from the IC method^28^.

#### Comparison of OCV-SOC curves derived from LC and IC methods

**Fig. 6:**
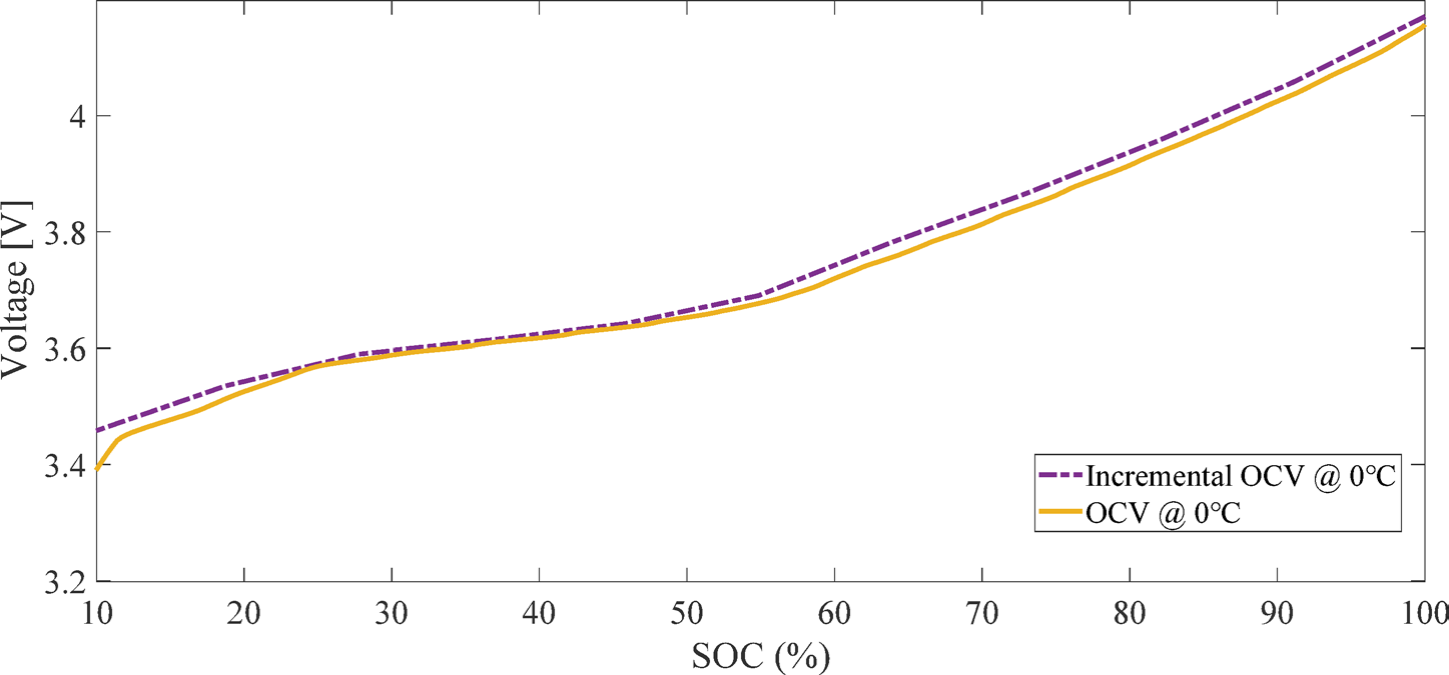
Comparison of OCV-SOC curves derived from the LC and IC methods.

Figure 6 compares the OCV-SOC curves obtained from the LC and IC methods. Although the differences between the two methods are marginal, the relative advantage of the IC method is evident due to its higher resolution in regions with steep OCV slopes^26^. In this study, both the LC and IC methods are employed for simulating SOC and R_0_ estimation, and their performance is evaluated by comparing the resulting outcomes. To distinguish between the ECM modeling approaches, the OCV-SOC curves derived from the LC and IC methods are designated as Estimator1 and Estimator2, respectively. Notably, for instantaneous OCV estimation in solving the model equations, interpolation and lookup table methods are utilized.

### Battery modeling using ECM

**Fig. 7:**
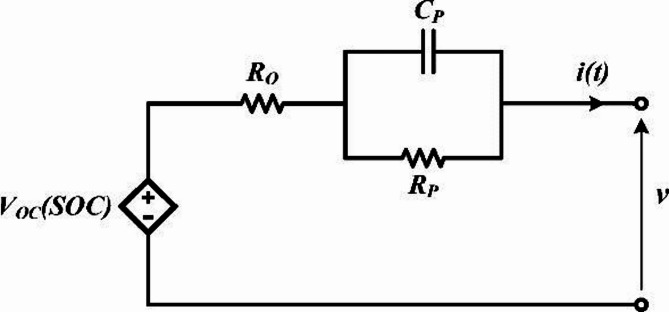
Equivalent circuit model of the battery.

For SOC estimation and SOH assessment, a simplified ECM with a single RC branch is employed (Fig. 7). This modeling choice represents a deliberate trade-off between computational efficiency and estimation accuracy, which is particularly relevant for real-time BMS applications. Although higher-order ECMs (e.g., 2RC models) can capture polarization dynamics more accurately under highly transient load profiles such as FUDS, they introduce additional parameters that increase model complexity, parameter coupling, and identifiability challenges within Kalman filter–based estimators. Since the primary objective of this study is to isolate and evaluate the impact of OCV–SOC modeling methods (LC versus IC) on UKF convergence behavior, SOC accuracy, and R_0_ tracking stability, the single-RC ECM is intentionally selected to avoid confounding effects arising from higher-order dynamics. This approach enables a clearer attribution of estimation performance differences to the OCV modeling strategy rather than to variations in model structure, while maintaining sufficient accuracy for dynamic operating conditions^18^. In this model, the open-circuit voltage as a function of the battery’s state of charge, V_OC_(SOC), is determined based on the OCV-SOC curves derived from the LC and IC methods, as described in the previous section.

The ECM can be described by the following first-order differential equations:

3 $$\:\frac{d}{dt}{v}_{c}\left(t\right)=\frac{-{v}_{c}\left(t\right)}{{R}_{p}{C}_{p}}+\frac{i\left(t\right)}{{C}_{p}}$$

4 $$\:v\left(t\right)=i\left(t\right){R}_{o}+{v}_{c}\left(t\right)+{v}_{oc}\left(soc\left(i\left(t\right)\right)\right)$$

5 $$\:soc\left(i\left(t\right)\right)=so{c}_{o}+\frac{\eta\:}{{C}_{bat}}\int\:i\left(t\right)d\left(t\right)$$

where *v*_*c*_*(t)* is the capacitor voltage, *v(t)* is the terminal voltage, SOC is the state of charge, *C*_*bat*_ is the battery capacity, *v*_*oc*_ is the open-circuit voltage, *i(t)* is the current, *η* is the coulombic efficiency, and *R*_*o*_, *R*_*p*_, and *C*_*p*_ are the electrical parameters of the ECM.

Since the battery capacity is estimated dynamically and continuously, the temperature dependence of the OCV-SOC characteristic is not considered in this model. Additionally, it is assumed that *R*_*o*_, *R*_*p*_, and *C*_*p*_ remain constant during ECM parameterization at a specific temperature (T), which may be the nominal temperature.

### SOC and ECM parameter Estimation using UKF

The UKF is employed for SOC and ECM parameter estimation, as it is well-suited for nonlinear systems^30^.

#### ECM parameter Estimation

The ECM parameters (***p****=[R*_*o*_,*R*_*p*_,*C*_*p*_*]*^*T*^) are estimated by solving an optimization problem. To this end, a discrete-time relationship is developed to predict the terminal voltage at the next time step, *v*_*k+1*_. The following equation is derived by solving for the capacitor voltage over a small time step *δ = ∆T* with the input current *i[k]* at step *k*:

6 $$\:{v}_{ck+1}={v}_{ck}{e}^{-\delta\:/{C}_{p}{R}_{p}}+{i}_{k}{R}_{p}\left(1-{e}^{-\delta\:/{C}_{p}{R}_{p}}\right)$$

Similarly, using the bilinear transformation, a discrete-time version of the SOC relationship is obtained:

7 $$\:so{c}_{k+1}=s\mathrm{o}{\mathrm{c}}_{k}+\frac{\eta\:.\delta\:}{2{C}_{bat}}\left({i}_{k}+{i}_{k-1}\right)$$

Additionally, the terminal voltage is defined as follows:

8 $$\:{v}_{k}={i}_{k}{R}_{o}+{v}_{ck}+{v}_{oc}\left(so{c}_{k}\right)$$

9 $$\:{v}_{k+1}={i}_{k+1}{R}_{o}+{v}_{ck+1}+{v}_{oc}\left(so{c}_{k+1}\right)$$

By substituting Eq. (6) into Eq. (9), we obtain:

10 $$\:{v}_{k+1}={i}_{k+1}{R}_{o}+{v}_{ck}{e}^{-\delta\:/{C}_{p}{R}_{p}}+{i}_{k}{R}_{p}\left(1-{e}^{-\delta\:/{C}_{p}{R}_{p}}\right)+{v}_{oc}\left(so{c}_{k+1}\right)$$

Finally, by solving for *v*_*c*_*[k]* from Eq. (8) and substituting into Eq. (10), we have:

11 $$\:{v}_{k+1}={i}_{k+1}{R}_{o}+\left({v}_{k}-{i}_{k}{R}_{o}-{v}_{oc}\left(so{c}_{k}\right)\right){e}^{-\delta\:/{C}_{p}{R}_{p}}+{i}_{k}{R}_{p}\left(1-{e}^{-\delta\:/{C}_{p}{R}_{p}}\right)+{v}_{oc}\left(so{c}_{k+1}\right)$$

The optimization problem for determining *R*_*o*_, *R*_*p*_ and *C*_*p*_ is formulated as follows:

12 $$\:\underset{p}{\mathrm{argmin}}\mathrm{:=}{\sum\:}_{k=1}^{N}\frac{1}{2}{\left({v}_{k+1}\left(p\right)-{v}_{k+1}^{\mathrm{*}}\right)}^{2}$$

where *v*^∗^_*k+1*_ is the actual measured terminal voltage at step *k + 1*. This optimization problem is solved using the Sequential Least Squares Programming (SLSQP) algorithm^27^.

#### SOC Estimation with UKF

After obtaining optimal coefficients for the OCV-SOC curve and ECM parameters, these values are used with the UKF to estimate SOC and predict the terminal voltage. The nonlinear state-space model is expressed as follows:

13 $$\:{x}_{k+1}=f\left({x}_{k},{i}_{k}\right)+{w}_{k},{\:\:y}_{k}=g\left({x}_{k}\right)+{u}_{k}$$

where $\:{x}_{k}={\left[\mathrm{so}{\mathrm{c}}_{k},{v}_{ck},{R}_{ok}\right]}^{T}$ is the state vector, $\:{w}_{k}$ is the process noise (with covariance *Q*_*w*_), *u*_*k*_ is the measurement noise (with covariance *R*_*u*_), *f(.)* is the nonlinear function predicting the next state *x*_*k+ 1*_ based on the current state *x*_*k*_ and *g(.)* is the observation function. The state and measurement equations are given by:

14 $$\:{x}_{k+1}={A}_{k}{x}_{k}+{i}_{k}{B}_{k}+{w}_{k}$$

15 $$\:{y}_{k}={i}_{k}{R}_{ok}+{v}_{ck}+{v}_{oc}\left(so{c}_{k}\right)+{u}_{k}$$

where:

16 $$\:{A}_{k}=\left[\begin{array}{ccc}1&\:0&\:0\\\:0&\:{e}^{-\delta\:/\tau\:}&\:0\\\:0&\:0&\:1\end{array}\right],{B}_{k}=\left[\begin{array}{c}\delta\:/{C}_{bat}\\\:{R}_{p}\left(1-{e}^{-\delta\:/\tau\:}\right)\\\:0\end{array}\right],{w}_{k}=\left[\begin{array}{c}{w}_{sock}\\\:{w}_{vck}\\\:{w}_{Rok}\end{array}\right]$$

In these equations, *C*_*bat*_ is the battery capacity, *δ* is the sampling time, and *τ* is the time constant (*τ = R*_*p*_*C*_*p*_). Sigma points in the UKF are used to approximate the state distribution, and the Unscented Transformation (*UT*) is employed to propagate the mean and covariance^26^.

### Application of real-world battery loading using the FUDS profile

To simulate real-world operating conditions, the FUDS profile is applied as a dynamic load^28^. The FUDS profile is selected for this analysis due to its diverse current variations. The current distribution in this profile is as follows:

- 55% of the time in discharge mode.
- 25% of the time in charge mode.
- 20% of the time in no-current (zero) mode.

**Fig. 8:**
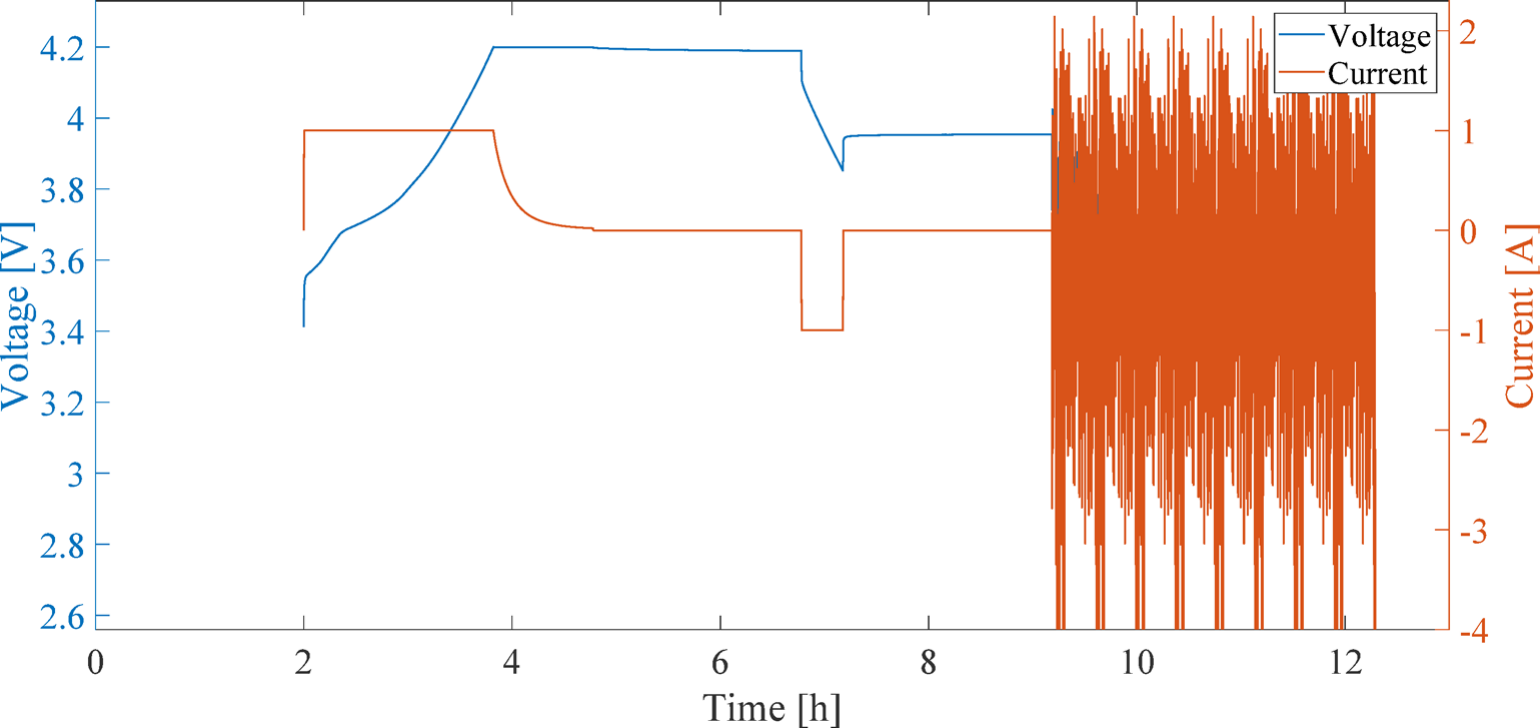
Current and voltage profiles measured during the comprehensive battery evaluation test.

**Fig. 9:**
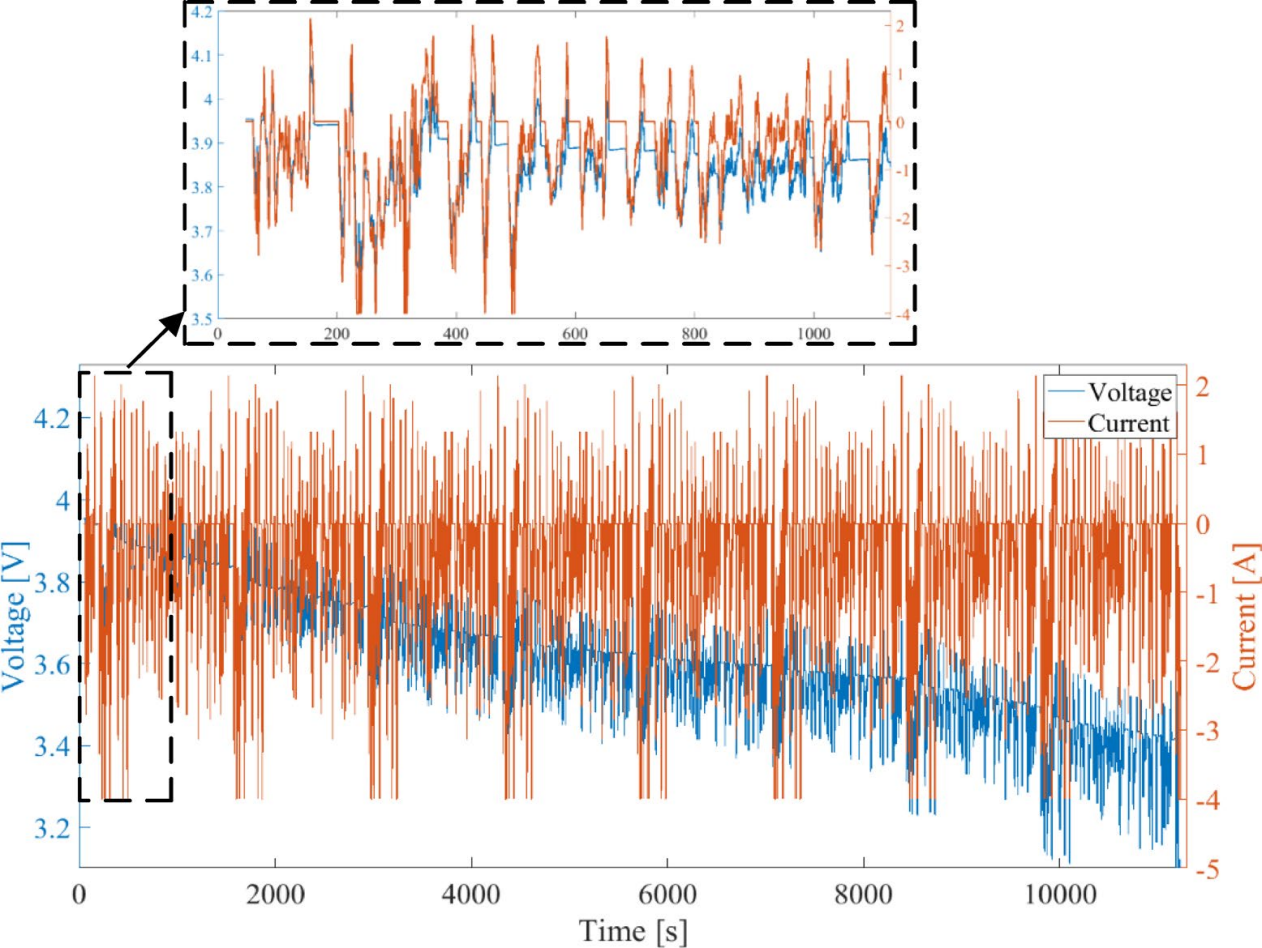
Current and voltage profiles measured under the FUDS dynamic loading profile at 80% of the nominal battery capacity (SOC₀ = 80%).

Figure 8 illustrates the current and voltage profiles measured during the comprehensive battery evaluation test. The test begins with an initial SOC of 80%, and the FUDS profile is applied after 9 h. Figure 9 presents the detailed current and voltage profiles under the FUDS profile. Due to its varied current changes, this profile provides quasi-realistic conditions for evaluating the model’s accuracy.

## SOC estimation results

### ECM parameter Estimation

The second step, after deriving the OCV-SOC curves using the LC and IC methods (designated as Estimator 1 and Estimator 2, respectively), is the estimation of the initial ECM parameters. These parameters include R_0_, Rₚ and Cₚ. The initial values for these parameters were selected based on experimental data provided in^24^, set at 0.07152, 0.01544, and 881.99, respectively. These values were assumed to be identical for both the LC and IC methods as a common starting point.

Subsequently, to achieve more accurate parameters and better alignment with the actual battery behavior, the UKF is employed alongside the solution of a least-squares optimization problem. This optimization is performed using the SLSQP algorithm, as described in Eq. (12), with the objective of minimizing the discrepancy between the model output and real-world data^27^. The UKF, utilizing measured observations, adaptively adjusts the model coefficients, ensuring rapid and stable convergence. Although the initial parameter values for Estimator 1 and Estimator 2 were set to be identical, slight differences were observed in their final values after optimization. These minor differences, despite being small, can significantly impact the accuracy of SOC and R_0_ estimation^29^.

### SOC estimation using UKF

**Fig. 10:**
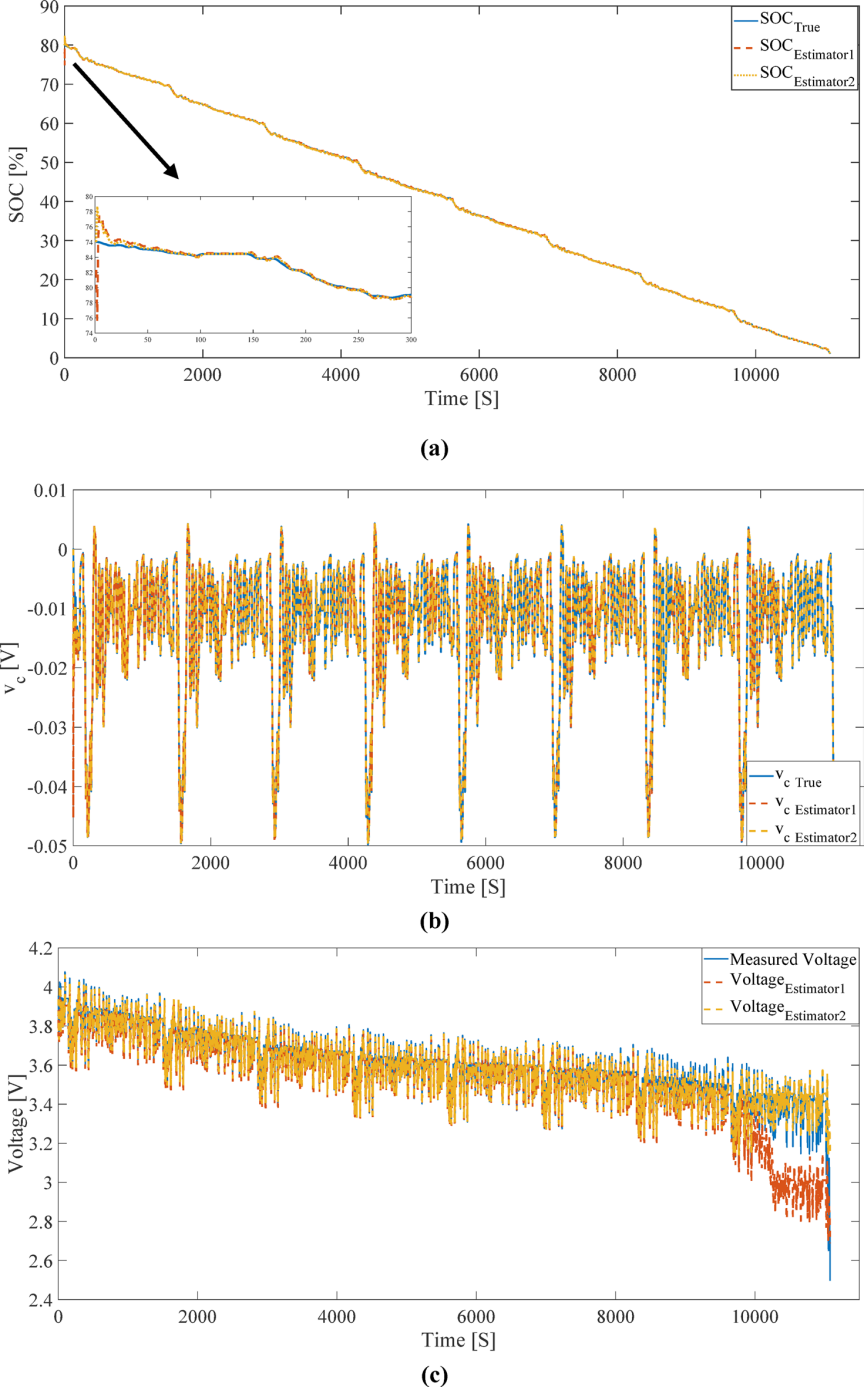
Estimation of (**a**) SOC, (**b**) *v*_*c*_, and (**c**) terminal voltage of the battery using the UKF and ECM under the FUDS profile with random initial noise.

The results of SOC, *v*_*c*_, and terminal voltage estimation, achieved by combining the ECM and the UKF, are presented in Fig. 10. In this simulation, FUDS test data from the publicly available CALCE dataset^28^ with an initial SOC of 80% were used. Figure 10 illustrates the estimation results assuming random initial noise in SOC. Here, the “true” values refer to those obtained from dynamic modeling (without estimation or noise), not experimental data. The results demonstrate that the UKF can accurately reconstruct the battery’s dynamic behavior and provide reliable estimates of the state variables.

The instantaneous open-circuit voltage values required by the measurement model are obtained using lookup tables derived from the LC and IC OCV–SOC curves. A piecewise linear interpolation scheme is employed to compute OCV values between discrete SOC breakpoints. This choice is motivated by its numerical robustness and widespread adoption in practical Battery Management Systems, where preserving local slope characteristics without introducing artificial oscillations is critical.

Unlike higher-order interpolation techniques such as spline interpolation, which may introduce overshoot or non-physical curvature in regions with steep OCV gradients, linear interpolation ensures monotonicity and stable sensitivity of the measurement function with respect to SOC. This property is particularly important for UKF-based estimation, as it prevents numerical instability and excessive gain amplification in high-slope regions of the OCV–SOC curve.

All simulations are conducted under fixed temperature conditions, consistent with the primary objective of this study, which is to isolate and evaluate the influence of OCV–SOC modeling strategies on UKF performance. While temperature variations are known to significantly affect internal resistance in real-world Electric Vehicle (EV) operating environments, explicitly incorporating electro-thermal dynamics would introduce additional state coupling effects that could obscure the comparative assessment between the LC and IC methods. Therefore, temperature-related influences are treated as quasi-static disturbances that are common to both estimators, ensuring a fair and controlled comparison.

It should be noted that the internal resistance R_0_ is inherently temperature-dependent. Accordingly, the results presented in this study reflect the relative robustness and stability of the LC and IC methods under identical thermal conditions rather than the absolute accuracy of resistance estimation under varying temperatures. Under practical EV operation, temperature-induced variations in R_0_ typically evolve more slowly than electrical dynamics, suggesting that the higher local sensitivity of the IC-based OCV model may offer improved resilience against such effects compared to the LC method.

To quantitatively evaluate the performance of the estimated parameters, the statistical metrics Mean Absolute Error (MAE) and Root Mean Square Error (RMSE) were calculated:

17 $$\:\mathrm{MAE}\mathrm{=}\frac{1}{N}{\sum\:}_{k=1}^{n}\left|{SOC}_{true,k}-{SOC}_{est,k}\right|$$

18 $$\:\mathrm{RMSE}\mathrm{=}\sqrt{\frac{1}{N}{\sum\:}_{k=1}^{n}{\left|{SOC}_{true,k}-{SOC}_{est,k}\right|}^{2}}$$

where SOC_ref, k_ *and* SOC_est, k_ represent the true and estimated values, respectively. Generally, a smaller MAE indicates better estimation performance. Compared to MAE, RMSE is more sensitive to large errors and is typically used to reflect variations in errors.

For quantitative evaluation of the UKF’s performance, the MAE, RMSE, and maximum error between the estimated and modeled values are presented in Table 1. The results indicate that Estimator 2 (based on the OCV derived from the IC method) outperforms Estimator 1 (based on the LC method) across all metrics. Specifically, for SOC estimation, the MAE for Estimator 2 is approximately one-third that of Estimator 1, and the maximum error is nearly one-sixth. This significant improvement stems from the steeper OCV slope in the IC method, which enhances measurement sensitivity to SOC variations, thereby increasing the filter’s correction gain.

The apparently higher maximum terminal voltage error observed for Estimator 2 originates from a very limited number of isolated outlier measurement points occurring toward the end of the FUDS profile, where the measured voltage exhibits abrupt deviations that are not consistent with the dynamic behavior captured by the equivalent circuit model. Owing to its higher local resolution in OCV–SOC modeling, Estimator 2 continues to follow the expected electrochemical response and does not reproduce these isolated anomalies, which results in a larger peak error despite superior overall accuracy. In contrast, Estimator 1 exhibits substantial voltage estimation errors over an extended time interval toward the final stage of the simulation, indicating a sustained mismatch under dynamic loading conditions that is more detrimental to long-term estimation reliability.

**Table 1:** Error metrics for Estimation under the FUDS profile with random initial noise.

Loading Type	Parameter	Error metric	Estimator 1 (LC)	Estimator 2 (IC)
FUDS	*v*	RMSE	0.122302	0.026551
		MAE	0.061906	0.013043
		Max error [V]	0.451182	0.623317
	SOC	RMSE	0.001272	0.001122
		MAE	0.000607	0.000709
		Max error [p.u.]	0.051878	0.034292
	*v*_*c*_	RMSE	0.001075	0.000761
		MAE	0.000427	0.000357
		Max error [V]	0.047940	0.034865

### UKF convergence analysis with initial SOC error

**Fig. 11:**
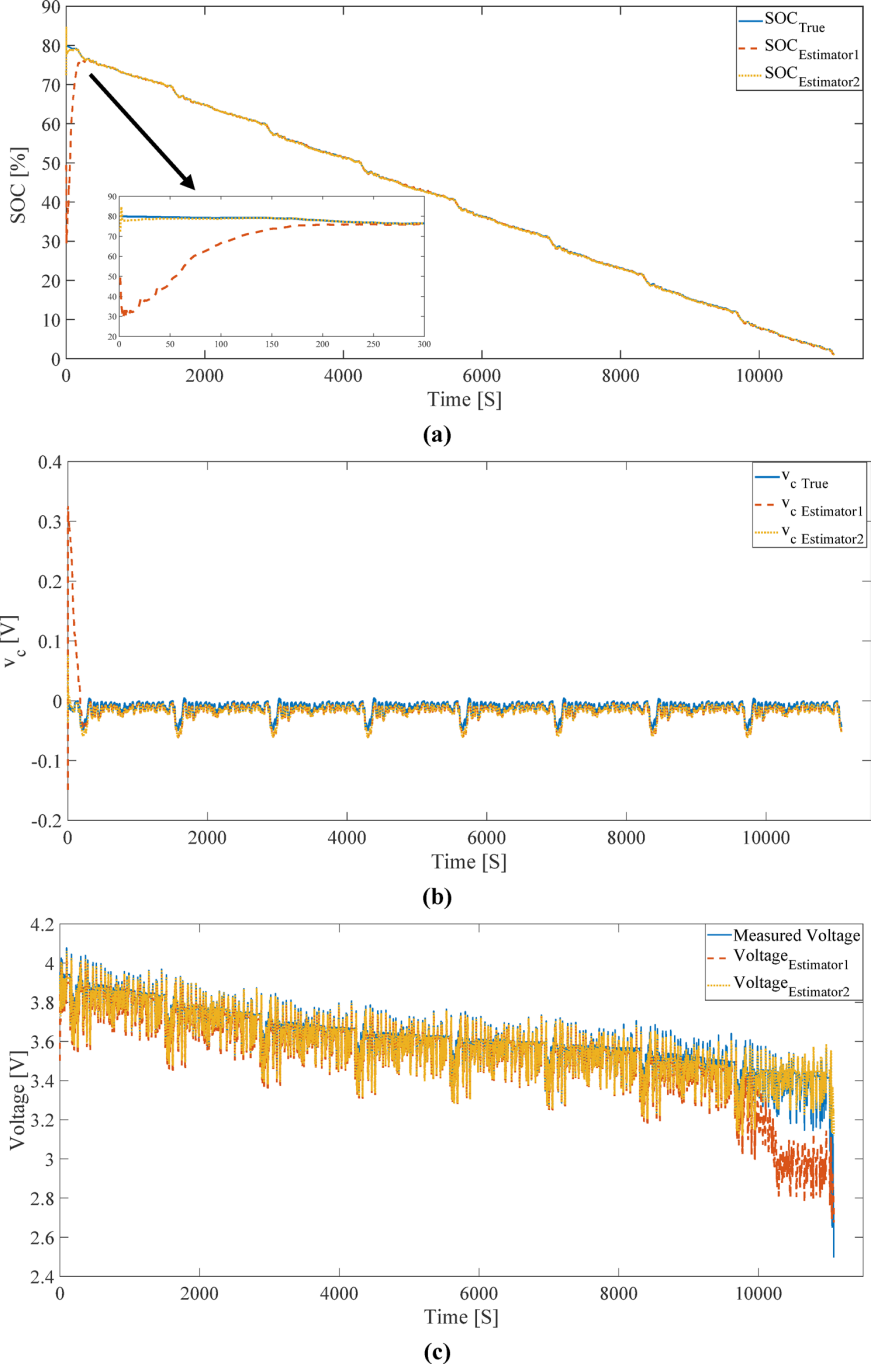
Estimation of (**a**) SOC, (**b**) *v*_*c*_, and (**c**) terminal voltage of the battery using the UKF and ECM under the FUDS profile with a 15% initial SOC error.

To evaluate the stability of the UKF against initial errors, the initial SOC was deliberately set with an error (0.65 instead of 0.80). The results of this simulation are presented in Fig. 11; Table 2. Despite the large initial error, Estimator 2 converged to the correct value in fewer than 10 time steps. In contrast, Estimator 1 required more than 200 time steps to reach the correct SOC value. This difference further underscores the importance of an accurate OCV model in the convergence of nonlinear filters.

This difference in convergence behavior can be further explained by examining the local sensitivity of the OCV–SOC relationship within the UKF measurement update. In nonlinear state estimation frameworks such as the UKF, the correction of SOC relies strongly on the local gradient of the OCV curve with respect to SOC (dV/dSOC), which determines how effectively voltage measurement residuals are translated into state corrections. When this gradient is small, SOC becomes weakly observable through voltage measurements, resulting in reduced correction capability despite the presence of measurement information.

Due to the averaging nature of the low-current experimental procedure, the LC-derived OCV–SOC curve exhibits reduced local voltage sensitivity over extended SOC ranges, including the operating region around the initial SOC level considered in this study. This reduced sensitivity leads to a diminished Kalman gain during the measurement update, thereby slowing the convergence of the SOC estimate when initial errors are present. In contrast, the IC method preserves finer local voltage variations induced by incremental current perturbations, yielding a more informative OCV–SOC mapping and consistently higher measurement sensitivity. As a result, the IC-based estimator achieves faster and more robust convergence of SOC under large initial errors, as observed in Fig. 11; Table 2.

From a practical implementation perspective, it is important to distinguish between the offline identification requirements and the online computational cost of the LC and IC approaches. Although the IC-based OCV–SOC model requires additional offline characterization steps—such as controlled current pulses and short rest periods during model development—these procedures are performed prior to deployment and do not impose any additional computational burden during online UKF operation. In real-time implementation, both LC and IC methods rely on equivalent lookup table evaluations and identical filter structures, resulting in comparable execution time and memory requirements. Therefore, the improved convergence robustness observed for the IC method is achieved without compromising real-time feasibility in production-level Battery Management Systems.

Additionally, the results indicate that, in both cases, the terminal voltage estimation remained largely unaffected by the initial SOC error. This stability arises from the measurement model structure, where changes in OCV due to SOC error are compensated by corresponding adjustments in *v*_*c*_ component. Consequently, the UKF was able to reconstruct the terminal voltage with high accuracy, even with a significant initial SOC error.

**Table 2:** Error metrics for Estimation under the FUDS profile with a 15% initial SOC error.

Loading Type	Parameter	Error Metric	Estimator 1 (LC)	Estimator 2 (IC)
FUDS	*v*	RMSE	0.123011	0.026959
		MAE	0.062382	0.013661
		Max error [V]	0.452531	0.623356
	SOC	RMSE	0.032119	0.001503
		MAE	0.003963	0.000676
		Max error [p.u.]	0.508828	0.075710
	*v*_*c*_	RMSE	0.024475	0.001355
		MAE	0.003184	0.000412
		Max error [V]	0.326631	0.076489

Analysis of the plots in Figs. 10(c) and 11(c) reveals that the terminal voltage estimation obtained from the LC method exhibits significant deviation from the true voltage and the IC method estimation in low SOC regions. This behavior directly results from the shape of the OCV function in the LC method, which has a low slope and reduced sensitivity to SOC changes in these regions. The low OCV slope prevents the UKF from effectively reflecting SOC variations in the measurement function, leading to a terminal voltage estimation that diverges from the true value.

In contrast, the IC method, by providing an OCV function with a steeper slope in critical regions, enhances the sensitivity of the estimation to SOC variations, enabling the UKF to correct SOC estimates more rapidly. Consequently, the terminal voltage estimated by the IC method maintains high agreement with the measured voltage, even in the presence of significant initial SOC error.

### Discussion and analysis

The impact of initial SOC error and the convergence behavior of the UKF to the true value were investigated. Simulation results demonstrated that, in the IC method, SOC estimation converged rapidly to the true value within a few initial time steps (fewer than 10 steps). In contrast, the LC method required over 200 time steps to achieve stable convergence. This discrepancy directly stems from the slope of the OCV function with respect to SOC in each method. The OCV function in the IC method typically exhibits a steeper slope, which enhances the sensitivity of the measurement function to SOC variations, thereby amplifying the filter’s gain in correcting the estimated value.

From the perspective of numerical stability, the UKF’s performance was observed to maintain terminal voltage estimation nearly unaffected, even in the presence of significant initial SOC error. This phenomenon arises from the composite structure of the measurement model, where changes in OCV due to SOC error are compensated by corresponding adjustments in the capacitor voltage component (*v*_*c*_). Consequently, the final estimated voltage exhibited good agreement with the measured voltage, and the initial SOC error had no significant impact on voltage accuracy.

From the perspective of filter robustness, this behavior indicates that the UKF is highly robust against initial SOC errors in battery systems. However, the type of OCV model can determine the speed of convergence and the quality of SOC estimation. Using models that provide richer information on SOC variations in voltage (such as the IC method) can enhance the filter’s performance under dynamic and noisy conditions.

From a modeling perspective, the terminal voltage estimation structure in the ECM is designed such that any error in OCV estimation due to SOC error must be compensated through the dynamic component (*v*_*c*_) or ohmic resistance. However, in the LC method, due to its inability to accurately represent battery behavior in low SOC regions, this compensation is not effectively achieved, leading to a significant loss of accuracy in terminal voltage estimation. This deviation is clearly visible in the plots of Figs. 10(c) and 11(c).

From a methodological standpoint, the present framework also opens several directions for future research. In particular, adaptive UKF formulations that dynamically adjust process and measurement noise covariances based on operating conditions could further improve robustness under highly transient load profiles and aging-induced parameter drift. In addition, hybrid OCV–SOC modeling strategies that combine the global smoothness of LC-derived curves with the enhanced local sensitivity of IC-based models may provide an effective trade-off between numerical stability and SOC observability. Such hybrid approaches could be especially beneficial for wide-SOC-range operation, where maintaining consistent estimation performance remains challenging in practical BMS.

Summary:

- The UKF is numerically stable and performs robustly against initial SOC errors.
- An OCV model with a steeper slope (IC method) enhances correction gain, reduces convergence time, and improves the UKF’s performance in accurate and rapid SOC estimation.
- Using an OCV model based on the IC method ensures numerical stability in terminal voltage estimation.

These results highlight the critical importance of selecting an appropriate OCV model for implementing state estimation filters in lithium-ion battery systems. Future work will include experimental validation using laboratory test benches or hardware-in-the-loop BMS platforms to evaluate the robustness of the proposed framework under real-world measurement noise, temperature variation, and sensor uncertainty. Such validation will further assess the practical applicability of the proposed OCV modeling comparison in real battery management systems.

## SOH Estimation via R_0_

One of the common methods for online SOH estimation of batteries is based on monitoring their internal resistance R_o_^9^:

19 $$\:\mathrm{SOH=}\frac{{R}_{\mathrm{o-old}}-{R}_{o-now}}{{R}_{\mathrm{o-old}}-{R}_{o-new}}\times\:100\mathrm{\%}$$

Here, R_o-new_ is the internal ohmic resistance of a new battery (at the factory), R_o-now_ is the current internal ohmic resistance, and R_o-old_ is the internal resistance at the end-of-life threshold (i.e., when the capacity reaches 80% of its initial value). To evaluate SOH, R_o-now_ must be obtained through modeling under real-world battery loading conditions. For this purpose, after modeling with the Kalman filter and the ECM, R_0_ can be calculated using the following relationship:

20 $$\:{R}_{o}\left(t\right)\mathrm{=}\frac{{v}_{meas}\left(t\right)-{v}_{oc}\left(soc\left(i\left(t\right)\right)\right)-{v}_{c}\left(t\right)}{i\left(t\right)}$$

Thus, R_0_ can be computed online and substituted for R_o-now_ in Eq. (19).

To analyze the long-term trend of R_0_ and mitigate the effects of noise and instantaneous fluctuations due to rapid current changes in the FUDS profile, a combination of linear regression and a moving average filter is employed^29^. The linear regression model is defined as follows:

21 $$\:{R}_{o}\left(t\right)\mathrm{=}a\left(t\right)+b$$

where:

- R_0_(t) is the estimated internal resistance at time (t),
- a is the slope of the regression line, indicating the rate of increase or decrease in resistance,
- b is the initial resistance value (intercept).

**Fig. 12:**
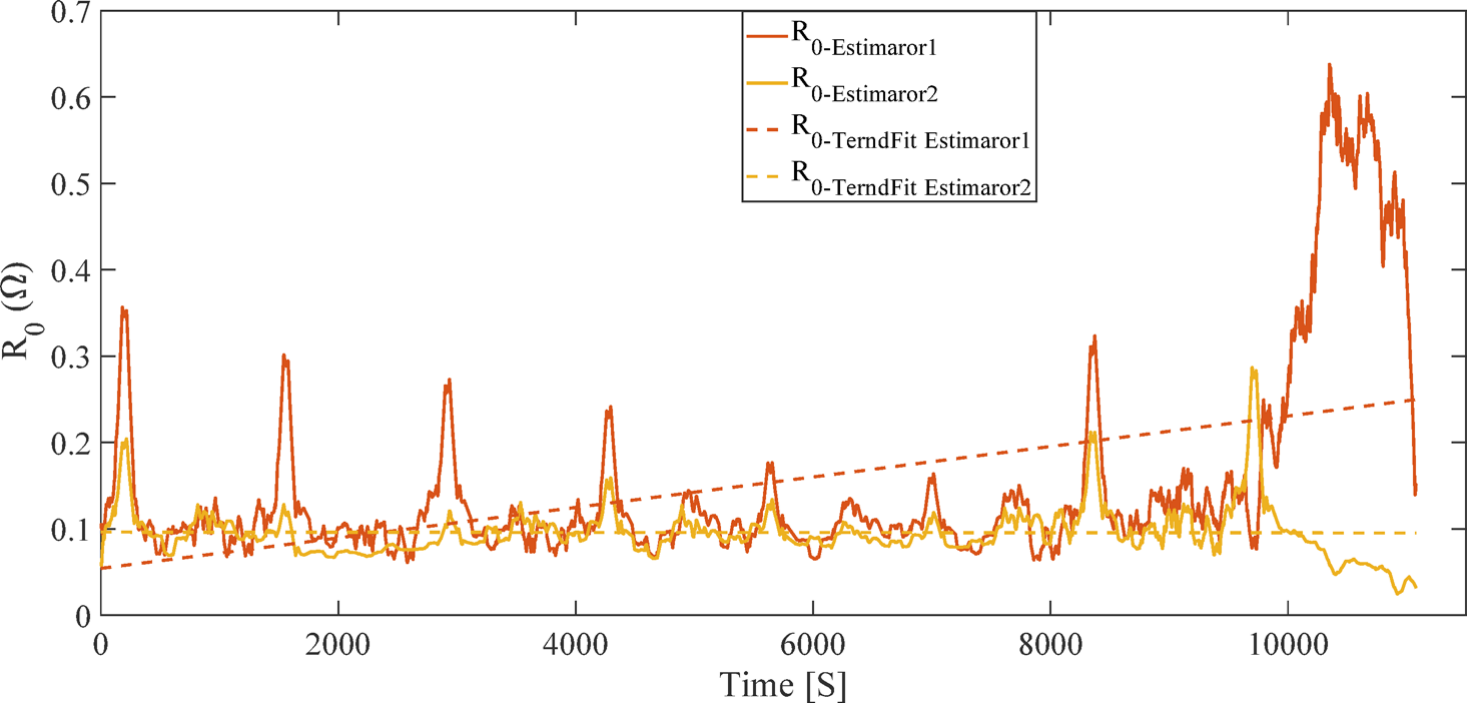
Analysis of R_0_ using two OCV estimation methods.

Linear regression was calculated by minimizing the sum of squared errors to determine the overall trend of R_0_ variations (e.g., increase due to battery aging). Additionally, a moving average filter was applied to smooth the R_0_ data and reduce noise caused by dynamic current and voltage variations. This filter, by averaging R_0_ values over a specified time window, more clearly revealed the long-term behavior of the internal resistance. The analysis results of the R_0_ trend for Estimator 1 and Estimator 2 are presented in Fig. 12.

Figure 12 shows that Estimator 2 exhibits fewer fluctuations in R_0_ estimation compared to Estimator 1, offering greater numerical stability. This advantage is due to the higher accuracy of the OCV-SOC curve in the IC method, which demonstrates lower sensitivity to transient current disturbances and rapid SOC changes^26^. In contrast, the LC method, due to its assumption of near-zero current in OCV derivation, experiences sudden jumps in R_0_ estimation under high instantaneous currents (such as in the FUDS profile).

The regression line slope for Estimator 2, at 2.2853e-08 Ω/sample, is significantly lower than that for Estimator 1, at 1.7106e-05 Ω/sample, indicating a more stable and realistic R_0_ trend estimation in the IC method. The higher slope in the LC method may be attributed to an overestimation of the resistance increase rate due to its sensitivity to noise^30^. These differences confirm that selecting the IC method for OCV-SOC derivation can significantly improve the accuracy and stability of SOH estimation.

The pronounced fluctuations observed in the estimated internal resistance R_0_ for Estimator 1, as shown in Fig. 12, primarily originate from numerical sensitivity rather than abrupt physical changes in battery impedance. In the adopted ECM–UKF framework, R_0_ is indirectly inferred through the voltage measurement residual, which is strongly coupled to SOC estimation accuracy. When the OCV–SOC curve exhibits reduced local sensitivity, as in the LC method, small voltage mismatches can be disproportionately attributed to variations in R_0_, leading to amplified short-term oscillations in its estimated value.

From a practical BMS perspective, such fluctuations may have important implications. Although modern BMS implementations typically apply temporal filtering, rate limiters, or hysteresis thresholds to resistance-based SOH indicators, persistent or high-amplitude oscillations in R_0_ could potentially lead to false End-of-Life (EOL) flags or premature degradation alarms if resistance is used directly as a health metric. In contrast, the IC-based estimator produces a smoother and more physically consistent R_0_ trajectory by improving SOC observability and reducing measurement ambiguity, thereby lowering the risk of false SOH or EOL indications under dynamic operating conditions.

Furthermore, the results showed that the initial SOC error (e.g., the 15% deviation examined in Sect. 3.3) had a negligible impact on R_0_ estimation. This stability is due to the rapid convergence of the UKF, which corrects initial errors within a few time steps. The moving average filter also improved the long-term accuracy of R_0_ estimation by reducing numerical fluctuations, a critical feature for battery health monitoring in practical applications such as electric vehicles^23^.

## Conclusion

This study investigated the impact of OCV-SOC curve derivation methods (based on the LC and IC methods) on the performance of the UKF in simultaneously estimating SOC and SOH of lithium-ion batteries. The results demonstrated that the IC method, due to its higher resolution in regions with steep OCV-SOC curve slopes, provides faster convergence and lower error compared to the LC method. This superiority was also observed in the estimation of R_0_, where the IC method exhibited fewer fluctuations and a more stable regression slope, yielding a more accurate and realistic representation of battery aging trends. The findings confirmed that the UKF is robust against initial SOC errors, achieving high numerical stability in terminal voltage estimation by compensating for OCV variations through the *v*_*c*_ component. The use of a moving average filter effectively reduced numerical fluctuations in R_0_ estimation, a critical feature for real-time battery health monitoring in applications such as electric vehicles and energy storage systems. This study provides an integrated framework for evaluating the impact of OCV derivation methods on the accuracy of SOC and SOH estimation, contributing to improved BMS efficiency and extended lithium-ion battery lifespan. These results offer practical guidance for developing advanced BMS solutions in industrial applications. Moreover, the enhanced convergence and robustness of the IC-based estimator are obtained without increasing the online computational complexity, as discussed in Sect. 3.3.

## Data Availability

The datasets used in this study are publicly available:- FUDS profile: CALCE Battery Data Repository [28].- NASA Battery Dataset: NASA Prognostics Data Repository [19].- All simulation codes are available from the corresponding author upon reasonable request.
